# Lestaurtinib Inhibits Histone Phosphorylation and Androgen-Dependent Gene Expression in Prostate Cancer Cells

**DOI:** 10.1371/journal.pone.0034973

**Published:** 2012-04-20

**Authors:** Jens Köhler, German Erlenkamp, Adrien Eberlin, Tobias Rumpf, Inna Slynko, Eric Metzger, Roland Schüle, Wolfgang Sippl, Manfred Jung

**Affiliations:** 1 Albert-Ludwigs-University Freiburg, Institute of Pharmaceutical Sciences, Albertstrasse, Freiburg, Germany; 2 Department of Pharmaceutical Chemistry, Martin-Luther University of Halle-Wittenberg, Halle/Saale, Germany; 3 Department of Urology/Women's Hospital and Center for Clinical Research, University of Freiburg Medical Center, Freiburg, Germany; Florida International University, United States of America

## Abstract

**Background:**

Epigenetics is defined as heritable changes in gene expression that are not based on changes in the DNA sequence. Posttranslational modification of histone proteins is a major mechanism of epigenetic regulation. The kinase PRK1 (protein kinase C related kinase 1, also known as PKN1) phosphorylates histone H3 at threonine 11 and is involved in the regulation of androgen receptor signalling. Thus, it has been identified as a novel drug target but little is known about PRK1 inhibitors and consequences of its inhibition.

**Methodology/Principal Finding:**

Using a focused library screening approach, we identified the clinical candidate lestaurtinib (also known as CEP-701) as a new inhibitor of PRK1. Based on a generated 3D model of the PRK1 kinase using the homolog PKC-theta (protein kinase c theta) protein as a template, the key interaction of lestaurtinib with PRK1 was analyzed by means of molecular docking studies. Furthermore, the effects on histone H3 threonine phosphorylation and androgen-dependent gene expression was evaluated in prostate cancer cells.

**Conclusions/Significance:**

Lestaurtinib inhibits PRK1 very potently in vitro and in vivo. Applied to cell culture it inhibits histone H3 threonine phosphorylation and androgen-dependent gene expression, a feature that has not been known yet. Thus our findings have implication both for understanding of the clinical activity of lestaurtinib as well as for future PRK1 inhibitors.

## Introduction

Epigenetics is defined as inheritable changes in gene regulation that are not determined by alterations in the genome [1]. Epigenetic processes have clear implications for the pathology of human disease [2], and hence new inhibitors of these are highly interesting for drug discovery [3]. Among diverse histone modifications [4], phosphorylation of histones is not so well studied, especially with regard to drug discovery. Most reports are on Aurora kinases which are rather involved in the control of mitosis [5]. Another kinase involved in mitosis that is acting on histones is haspin [6], [7]. The kinases PKC-betaI [8] and PRK1^a^ (also termed PKN1) [9] play important roles in activating gene transcription [10] in the course of androgen receptor signalling and PRK1 is considered to be a promising target for the treatment of prostate cancer. In the search for new PRK1 inhibitors we performed a focussed library screening to identify new hits and evaluate reference kinase inhibitors in comparison. We identified the clinical candidate lestaurtinib (also known as CEP-701) as a new potent inhibitor of the epigenetic kinase PRK1.

## Results

### Focused Library Screening

**Figure 1:**
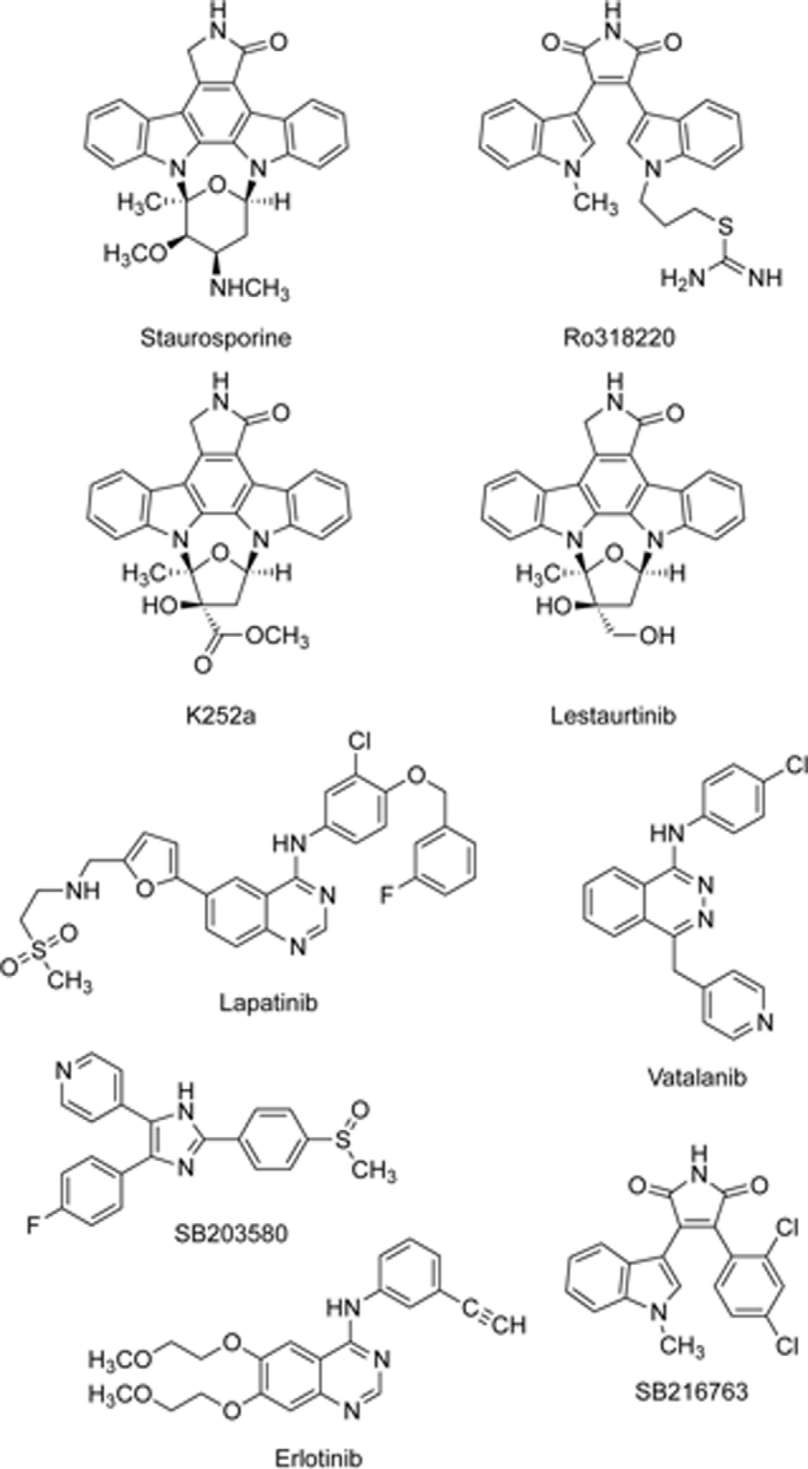
Known and newly identified PRK1 inhibitors.

As a starting point for the search of new PRK1 inhibitors, we used the Biomol Kinase and Phosphatase inhibitor library (n = 84, see Figure S4, S5 and S6) for an initial screening at 100 nM threshold concentration. This screening identified only the bisindolyl-maleimide (BIM) Ro318220 and the structurally related staurosporine as hits (more than 40% binding relative to staurosporine at 100 nM) (see Figure 1 and Table 1). Ro318220 was already known to inhibit PRK1 [9]. We further screened a 200 compound in-house library of commercially available and generic kinase inhibitors, resp. inhibitor candidates. Those included standard kinase inhibitors like erlotinib, lapatinib, vatalanib, SB203580 and SB216763 (see Figure 1), which have been used to profile different kinases before. The inhibitors K252a and lestaurtinib and additionally SB216763 (interaction data not shown) were selected for the docking study based on their structural similarity to staurosporine and Ro318220. The staurosporine analogs all show a similar binding model. K252a inhibits trkA, VEGFR2 and MLK1 in the two-digit nM region and is known to have a selectivity over PKC about 10-20fold [11]. Lestaurtinib was reported to inhibit trkA, B and C [12], JAK [13] and FLT3. Because of the inhibition of FLT3, it is studied clinically in myelofibrosis and AML [14], [15]. Lestaurtinib and K252a were both bound by PRK1 with high affinity (see Table 1). Lestaurtinib was chosen for further biological evaluation in our study due to its advanced development status and showed inhibition of androgen gene responsive gene transcription.

**Table 1:** PRK1 in vitro binding.

Inhibitor	IC50 ± SEM [nM]
Staurosporine	0.8 ± 0.2
Ro318220	28.6 ± 6.5
K252a	3.2 ± 0.6
Lestaurtinib	8.6 ± 0.9

### Molecular Modelling

**Figure 2:**
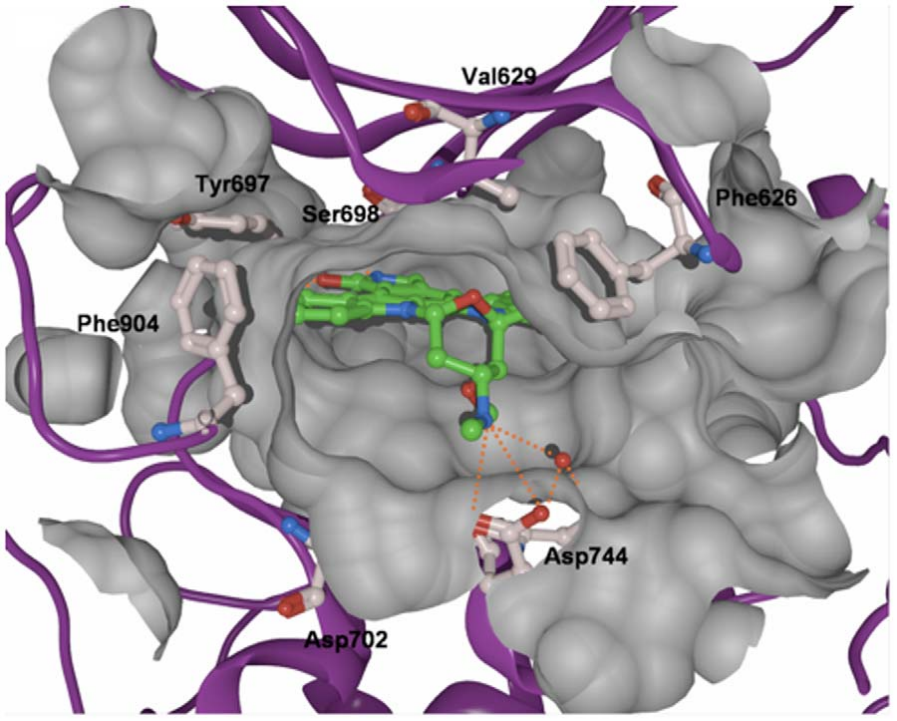
Interaction of staurosporine (carbon atoms in green) at the ATP binding pocket of PRK1. The molecular surface of the binding pocket is shown. Besides the hydrogen bonds with the hinge region, the protonated amino group of staurosporine donates hydrogen bonds (shown as orange dashed lines) to Asp744 and a conserved water molecule (shown in red).

A model of human protein C related kinase 1 (PRK1) was generated by homology modeling to rationalize our findings for further optimization studies. The model was based on similarity of PRK1 to protein kinase C theta (PKC-theta) and was used for docking studies. Since the available crystal structures of PKC subtypes in complex with inhibitors show all the active kinase conformation, the PRK1 model represents also the active conformation with the classical DFG-in motif (Figure 2) [16]. The 84 compounds of the Biomol kinase inhibitor library, which were tested in the in-vitro assay, were docked into the ATP binding pocket of PRK1. We used three different docking methods (GOLD [17], GLIDE [18] and ParaDockS [19]) and five different scoring functions (ChemScore, GoldScore, GlideScore, ParaDockS p-Score and AMBER GBSA score [20], [21]). In total, 63 compounds could be successfully docked into the ATP binding pocket. In general, the different docking methods yielded similar results even if the ranking of the compounds was found to be different (Table S1). The two active inhibitors from the Biomol compound collection, staurosporine and Ro318220, were top-ranked by some of the scoring functions. Generally, in docking and virtual screening studies a better discrimination between actives and decoys is observed by using a consensus score based on a variety of different scoring methods [22], [23]. In the present study, we also calculated a consensus score using the five normalized scoring functions Chemscore, Goldscore, Glidescore, ParaDockS p-score and GBSA score. Using the consensus scoring, a clear discrimination between the highly active inhibitors staurosporine (Score 9.23) and Ro318220 (Score 9.51) and the inactive compounds (including the inactive kinase inhibitors shown in Figure 1) could be obtained. The only molecule which gave a similar high score was the related bisindolylmaleimide derivative **31** (Score of 8.84, Table S1, Figure S5) which did not show any in-vitro activity below 100 nM (data not shown).

**Figure 3:**
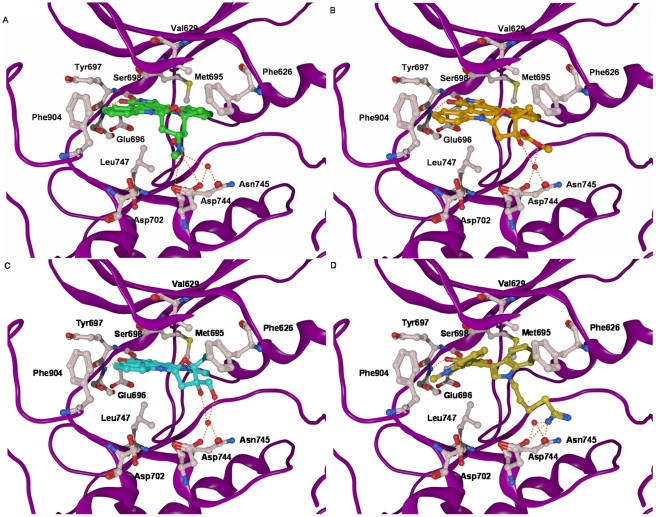
Details of the binding of (A) staurosporine (green), (B) K252a (orange), (C) lestaurtinib (cyan), and (D) Ro318220 (dark-yellow) to the PRK1 kinase domain. Common interactions of the inhibitors are hydrogen bonds involving Glu696 and Ser698 of the hinge region, and van-der-Waals interactions with the gatekeeper residue Met695, as well as with Val629, Phe626, Leu747, and Phe904. In addition, some of the inhibitors interact with Asp744, Asn745 and a conserved water molecule (red sphere) nearby the Mg^2+^ binding site of the kinase. The backbone is shown as a purple ribbon. Only relevant amino acids are displayed. Hydrogen bonds are shown as dashed orange colored lines.

The docking arrangement of staurosporine in the active site of PRK1 shows that the ligand is completely buried in the ATP binding pocket (Figure 2). Most important, the lactame and maleimide groups of staurosporine and Ro318220, respectively, form two hydrogen bonds to Glu696 and Ser698, which are part of the hinge region of PRK1 (Figure 3A and 3D). Van-der-Waals interactions are observed with the gatekeeper residue Met695, as well as with Val629, Phe626, Leu747, and Phe904, respectively. Furthermore, hydrogen bonds between the protonated amino group of staurosporine as well as the thiourea group of Ro318220 and Asp744 can be observed. The amino group of staurosporine is also involved in a hydrogen bond with a conserved water molecule and the backbone CO of Asp744.

The analysis of the docking poses of K252a and lestaurtinib showed that both compounds interact in the same way with the ATP binding pockets as staurosporine (Figure 3B and 3C). Besides the interaction with the hinge region, the polar substituents at the tetrahydrofuran ring system interact with a conserved water molecule bound at Asp744 and Asn745.

### Cellular Testing

**Figure 4:**
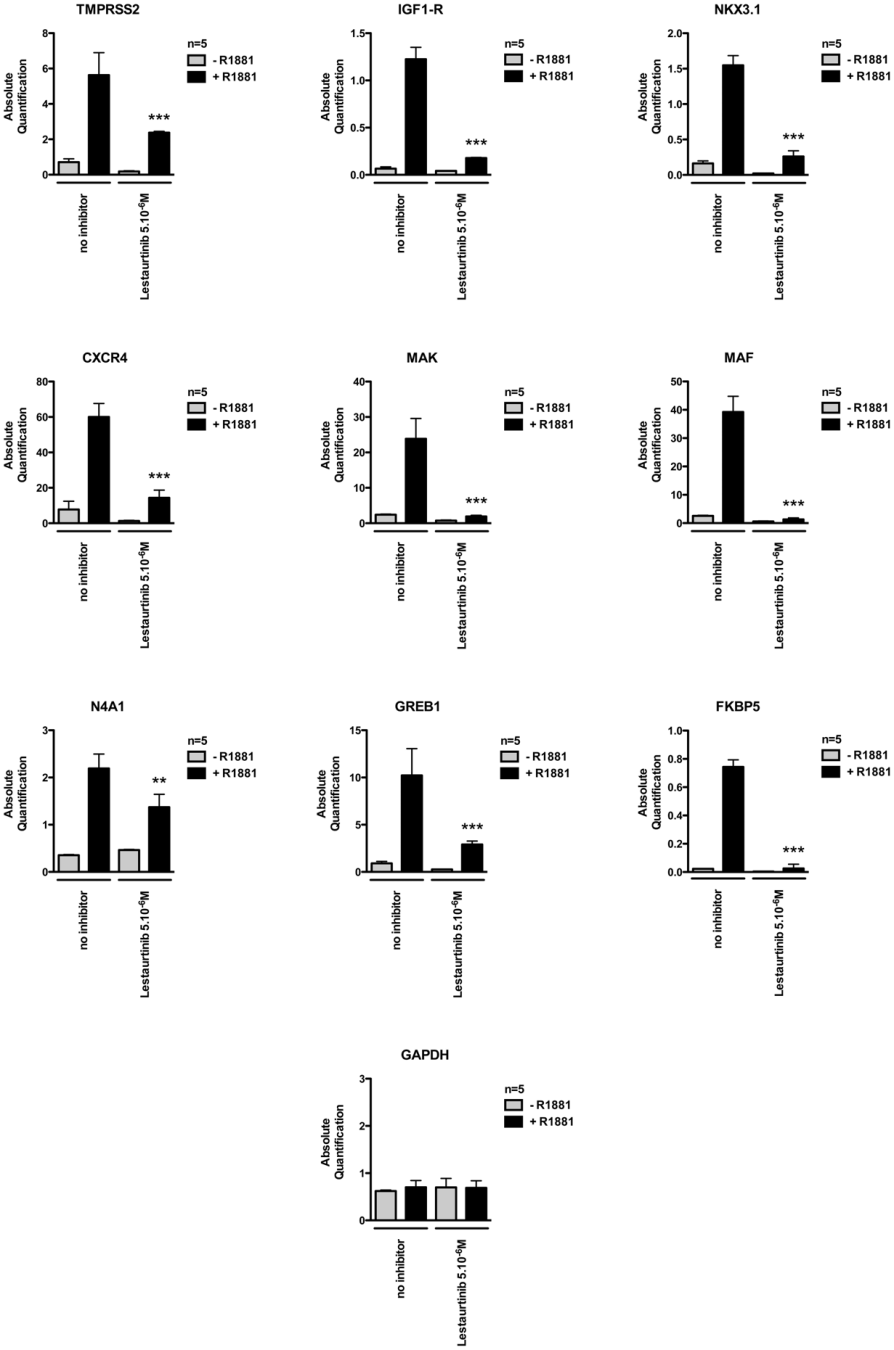
Effects of lestaurtinib on the mRNA expression of the androgen receptor target genes. Expression of androgen receptor target genes *TMPRSS2*, *IGF1-R*, *NKX3.1*, *CXCR4*, *MAK*, *MAF*, *N4A1*, *GREB1* and *FKBP5* measured by qRT-PCR in androgen (R1881) stimulated LNCaP prostate cancer cells are lowered by the treatment with lestaurtinib (final concentration 5 µM). The mRNA expression of the *GAPDH* gene was used as a control. Bars represent mean + SD (n = 5). P-value: ns  =  non significant; *  =  <0.05; **<0.01; ***<0.001.

We then analyzed the effect of lestaurtinib on androgen responsive LNCaP prostate cancer cells (see Figure 4) [24]. An effect on androgen receptor mediated signalling was measured using the quantitation of mRNA expression of several known androgen receptor target genes. Lestaurtinib blocks the expression of Transmembrane protease, serine 2 (*TMPRSS2)* [25], Insulin-like growth factor-1 (*IGF1-R27)* [25], CXC chemokine receptor 4 (*CXCR429)* [26], Homeobox protein Nkx-3.1 (*NKX3.1)* [27], male germ cell-associated kinase *(MAK30)* [28], musculoaponeurotic fibrosarcoma oncogene homolog (*MAF)* [*29*], nuclear receptor subfamily 4, group A, member 1 (*N4A1)* [29], Gene regulated in breast cancer I *(GREB1)* [30] and FK506 binding protein 5 (*FKBP5)* [31] at 5 µM but does not reduce expression of the androgen-independent glyceraldehyde-3-phosphate dehydrogenase (*GAPDH)* gene [31]. In the same range of concentration, lestaurtinib caused a hypophosphorylation at histone H3 threonine 11 (Figure S7).

## Discussion

Histone modifications have become a focus of drug discovery efforts. Inhibitors of the enzymes that establish these modifications are also valuable tools to probe signalling pathways. We identified the clinical candidate lestaurtinib as an inhibitor of the kinase PRK1 which affects epigenetic regulation and androgen receptor signalling. This new mode of action of lestaurtinib has not been known so far and could be important to understand its activity in clinical settings. The structural similarities of lestaurtinib in comparison to staurosporine make it possible that other kinases than PRK1 may contribute to the observed gene regulatory effects in LNCaP cells. Nevertheless, it is of great importance that lestaurtinib has an effect on epigenetic histone modifications and reduces androgen dependent gene expression significantly in prostate cancer cells. This fact should be taken into consideration for future assessments in clinical settings.

In addition to the in vivo effects of lestaurtinib, the identification of lestaurtinib and K252a as novel PRK1 inhibitors and the lack of inhibition of established kinase inhibitors with scaffolds such as anilinoquinazolines, anilinophthalazines or diarylimidazoles provides valuable information for the design of further and more selective PRK1 inhibitors as potential therapeutics for androgen-dependent cancers.

## Materials and Methods

### Kinase Assay

Kinase inhibitors were purchased from Sigma-Aldrich (Lestaurtinib) and Biomol (Ro318220) and used as obtained. Purity was >93% (w/w) in all cases as indicated by the supplier. Inhibitor screening was carried out with the LanthaScreen™ Eu Kinase Binding Assay Kit (Invitrogen) with final assay concentrations of 5 nM for PRK1 (Proqinase, Freiburg), 2 nM LanthaScreen Eu-anti-GST Antibody (Invitrogen) and 10 nM Kinase Tracer 236 (Invitrogen). The assay was performed in 384-well microtiter plates (Perkin Elmer, Rodgau). The final assay volume was 15 µL. Detection was performed with EnVision 2102 Multilabel Reader (PerkinElmer, Rodgau, Excitation: 340 nm, 1^st^ Emission: 665 nm, 2^nd^ Emission: 615 nm, Delay Time 100 µs, Integration Time 200 µs). The kinase inhibitor library was purchased from Biomol (academic size, n = 84 - former CAT#2831A, Figure S4, S5, S6). IC_50_ determinations were performed three independent times, each in triplicates.

### Computational Methods

Al0l calculations were performed on a Pentium IV 2.2 GHz based Linux cluster. Since the crystal structure of the human PRK1 kinase (Uniprot entry Q16512) is not known, a homology model was generated. A BLASTP search [32] was performed for the human PRK1 amino acid sequence. The highest similarity was found for the related kinase PKCtheta. The overall sequence identity between the human PRK1 sequence and the sequence derived from PKC-theta is 49% with only small gaps or insertions in the aligned region. The sequence alignment was done with ClustalW [33] (Figure S1). The model was generated with the program Modeller using the coordinates of the PKC-theta X-ray structure 2jed (Resolution 2.32 Å) in the active conformation. The superimposition of the PRK1 model and the PKC-theta X-ray structure yielded an RMSD value of 0.73 Å for the backbone atoms illustrating the high structural similarity of both kinases. The stereochemical quality of the resulting PRK1 model was analyzed using the program PROCHECK [34]. Hydrogen atoms and AMBER partial charges were assigned. The model was energy-minimized using the AMBER force field [35]. The refinement led to a model with excellent stereochemical quality with 90.1% of the phi and psi angle values in the most favored regions, and 8.5% in additionally allowed regions (Figure S2). No residue in the model is located in the disallowed region.

### Ligand Docking

We used three different docking programs (GOLD 4.1 [17], GLIDE [18] and ParaDockS [19]) to dock all molecules into the PRK1 ATP binding site. For all docking runs the default settings of the program were used. The binding site for PRK1 was defined on Glu696 with a radius of 15 Å covering the ATP binding pocket. A conserved water molecule observed in the X-ray structures of PKCbeta and PKCtheta was considered to be part of the protein. Goldscore, Chemscore, Glidescore, ParaDockS p-Score and AMBER GBSA [20], [35] score were chosen as fitness functions. For each molecule, 30 docking runs were performed. The resulting solutions were clustered on the basis of the heavy atom RMSD values (1 Å). First, we tested whether the docking programs are able to reproduce the binding mode of the bound ligands NVP-XAA228, Biomol **33 (**Ro318220), and staurosporine as observed in the X-ray structures of PKC-theta and PKC-beta, respectively (pdb codes 2jed.pdb, 1xjd.pdb [36] and 1i0e.pdb [37]). Highest accuracy was obtained using the GOLD program and Goldscore as fitness function. The RMSD values between the top-ranked Goldscore docking solution and the crystal structure of the inhibitors are 0.78 Å, 0.71 Å and 0.49 Å (heavy atoms) respectively, demonstrating the usability of the applied docking programs for reproducing the experimentally determined structures of the kinase inhibitor complexes.

### Molecular Dynamics Simulations

The stability of the derived PRK1-inhibitor complexes was examined by means of molecular dynamics (MD) simulations. The most active inhibitor staurosporine was used for this simulation. MD simulations were carried out using AMBER 10 and the AMBER 1999 force field [38], [39]. The ligand force fields parameters were taken from the general Amber force field (GAFF), whereas AM1 ESP atomic partial charges were assigned to the inhibitor. The complexes were soaked in a box of TIP3P water molecules with a margin of 10 Å. Prior to the free MD simulations, two steps of relaxation were carried out; in the first step, we kept the protein fixed with a constraint of 500 kcal mol^−1^Å^−1^. In the second step, the inhibitor structures were relaxed for 0.5 ps, during which the protein atoms were restrained to the X-ray coordinates with a force constant of 500 kcal mol^−1^Å^−1^. In the final step, all restraints were removed and the complexes were relaxed for 1 ps. The temperature of the relaxed system was then equilibrated at 300 K through 20 ps of MD using 2 fs time steps. A constant volume periodic boundary was set to equilibrate the temperature of the system by the Langevin dynamics [40] using a collision frequency of 10 ps^−1^ and a velocity limit of 5 temperature units. During the temperature equilibration routine, the complex in the solvent box was restrained to the initial coordinates with a weak force constant of 10 kcal mol^−1^Å^−1^. The final coordinates of the temperature equilibration routine (after 20 ps) were then used to complete a 1 ns molecular dynamics routine using 2 fs time steps, during which the temperature was kept at 300 K by the Langevin dynamics using a collision frequency of 1 ps^−1^ and a velocity limit of 20 temperature units. The pressure of the solvated system was equilibrated at 1 bar at a certain density in a constant pressure periodic boundary by an isotropic pressure scaling method employing a pressure relaxation time of 2 ps. The time step of the free MD simulations was 2 fs with a cutoff of 9 Å for the non-bonded interaction, and SHAKE [41] was employed to keep all bonds involving hydrogen atoms rigid. Electrostatic interactions were computed using the Particle Mesh Ewald method [42]. The MD simulation of the PRK1-staurosporine complex was performed in total for 10 ns. The RMSD plot is shown in Figure S3.

### Database

The 3D structures of the Biomol compounds (Figure S4, S5, S6) were generated using the Omega program (OpenEye Software). All possible isomers and tautomers were generated, which resulted in 265 3D structures. All generated isomers were used for the docking study.

### AMBER GBSA Scoring

The docked poses were energy-minimized using the AMBER force field and the GBSA continuum model [21]. For each molecule the best scoring pose was selected for comparison with the biological data. The minimization was carried out using a combination of steepest descent and conjugate gradient algorithm with a root mean square of gradient at 0.001 kcal/mol. AM1-BCC charges were assigned for the inhibitors, and the Amber99 force field was applied for the protein. The non-bonded cutoff was set at 16 Å. All heavy atoms of the protein were tethered with a force constant of 100 kcal/mol, whereas the inhibitor atoms were relaxed during the energy minimization process. The binding free energy Δ*G* is calculated as:
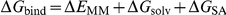
where Δ*E*_MM_ is the difference in energy between the complex and the sum of the energies for the free ligand and free protein, Δ*G*_solv_ is the difference in the GBSA solvation energy of the complex and the sum of the solvation energies for free ligand and free protein, and Δ*G*_SA_ is the difference in the surface area energy of the complex and the sum of the surface area energies for free ligand and free protein.

### RT-qPCR Analysis

Effects on androgen receptor target genes were determined using real-time quantitative PCR. LNCaP cells were washed once with PBS and then starved for 24 hours in phenol-red-free RPMI1640 supplemented with 0.5% double-stripped fetal calf serum (dsFCS). Cells were then treated or not with lestaurtinib (final concentration 5 µM) as indicated. If cells were not treated with lestaurtinib, DMSO was added as a vehicle. 10 minutes after adding lestaurtinib or the vehicle, cells were treated overnight with or without R1881 (10^−9^ M) as indicated. DNaseI-treated RNA, isolated using Trizol (Invitrogen), was used for reverse transcription. Quantitative PCR was performed in a LightCycler 480 (Roche). Product formation was detected by incorporation of SYBR green I using ROX as a passive reference (ABgene). For qRT-PCR, the following primers were used: *TMPRSS2* [25] 5′-TCACACCAGCCATGATCTGT-3′ and 5′-CTGTCACCCTGGCAAGAATC-3′; *IGF1-R* [25] 5′-CTGTATGCCTCTGTGAACC-3′ and 5′-TAGACCATCCCAAACGAC-3′; *NKX3.1* [27] 5′-AGAACGACCAGCTGAGCAC-3′ and 5′-AAGACCCCAAGTGCCTTTCT-3′; *CXCR4* [26] 5′-CTGTGAGCAGAGGGTCCAG-3′ and 5′-ATGAATGTCCACCTCGCTTT-3′; *MAK* [28] 5′-TGGACTTGCAAGAGAATTAAGGT-3′ and 5′-CTTCAGGGGCACGATACC-3′; *MAF* [29] 5′-AGCGGCTTCCGAGAAAAC-3′ and 5′-GCGAGTGGGCTCAGTTATG-3′; *N4A1* [29] 5′-ACAGCTTGCTTGTCGATGTC-3′ and 5′-GGTTCTGCAGCTCCTCCAC-3′; *GREB1* [30] 5′-AAGCTGAGCAGCACAGACAA-3′ and 5′-GGCTTCTCTTCTCCGAGGTAG-3′; *FKBP5* [31] 5′-TTTTTGAGATTGAGCTCCTTGA-3′ and 5′- TTGTGTTCACCTTTGCCAAC-3′; *GAPDH* [31] 5′-GAGTCCACTGGCGTCTTCAC-3′ and 5′-GTTCACACCCATGACGAACA-3′. Bars represent mean + SD (n = 5). P-value: ns  =  non significant; *  =  <0.05; **<0.01; ***<0.001.

### Western Blot Analysis

LNCaP human prostate adenocarcinoma cells were grown in RPMI1640 medium (PAA) containing fetal bovine serum (PAA) 10% (v/v), penicillin (PAA) 1% (v/v), streptomycin (PAA) 1% (v/v), L-glutamine (PAA) 1% (v/v) at 37°C and 5% CO_2_. 2.5 * 10^6^ cells were seeded in tissue culture dishes (10 cm) and incubated in RPMI1640 medium (PAA) containing charcoal stripped fetal calf serum (PAA) 10% (v/v), penicillin (PAA) 1% (v/v), streptomycin (PAA) 1% (v/v), L-glutamine (PAA) 1% (v/v). 24 h after seeding lestaurtinib or Ro318220 (final concentration in each case 4.5 µM) dissolved in growth medium (charcoal stripped bovine serum 10% (v/v), penicillin 1% (v/v), streptomycin 1% (v/v), L-glutamine 1% (v/v), DMSO 1% (v/v), dihydrotestesterone 0.1 nM) were added and incubated for 18 h. LNCaP cells incubated with DMSO (1% (v/v)) were used as a control. Following inhibitor treatment, cells were washed once with ice cold PBS, lysed with nuclei isolation buffer (Sucrose 250 mM, Tris-HCl 20 mM, NaCl 150 mM, IGEPAL-CA 630 0.1% (v/v), CaCl_2_ 0.2 mM, MgCl_2_ 1.5 mM, DTT 1 mM, Pepstatin 1 µM, Protease Inhibitor Cocktail Mix (Sigma), PhosStop® (Roche), pH 8.0). Histones were acid extracted (H_2_SO_4_ 0.4 N, DTT 1 mM, Pepstatin 1 µM, Protease Inhibitor Cocktail Mix (Sigma), PhosStop® (Roche)) overnight.

Protein concentrations of histone extracts were determined using BCA Protein Assay (Pierce). 5 µg of proteins were separated by SDS-gel electrophoresis (15% polyacrylamide gel) and transferred to a Roti®-PVDF membrane (Roth). The membrane was then blocked with non-fat dry milk (Roth, 5% (w/v), TBS, Tween 20 0.1% (v/v)) and probed with anti-H3T11ph (Active Motif) 1∶1000 and anti-H3 (Upstate) 1∶5000 as a loading control. Canon CanoScan Lide 50 was used to acquire the image of the blot and Adobe Photoshop 5.0 was used to process the image.

## Supporting Information

Figure S1 **Sequence alignment between PKC-theta (Sbjct) and PRK1 (Query).**
(TIFF)

Figure S2 **PROCHECK analysis.**
(TIFF)

Figure S3 **RMSD plot of the MD simulation of the PRK1-staurosporine complex using AMBER10.** The grey line represents the RMSD plot for the PRK1 protein Cα-atoms, whereas the black line shows the fluctuation of the inhibitor structure.
(TIFF)

Figure S4 **Compounds from the Biomol library, Compounds 1–30.**
(TIFF)

Figure S5 **Compounds from the Biomol library, Compounds 31–60.**
(TIFF)

Figure S6 **Compounds from the Biomol library, Compounds 61–84.**
(TIFF)

Figure S7 **Effects of lestaurtinib and Ro318220 on the phosphorylation of H3T11 in LNCaP cells after 18 h.**
(TIFF)

Table S1 **Scoring values obtained for 63 docked Biomol compounds and seven more of the tested kinase inhibitor.**
(DOC)
