# Supplementary figures and images for: Lestaurtinib Inhibits Histone Phosphorylation and Androgen-Dependent Gene Expression in Prostate Cancer Cells
Source: PLoS One. 2012 Apr 20;7(4):e34973. doi: 10.1371/journal.pone.0034973 (PMC3332061; doi:10.1371/journal.pone.0034973)

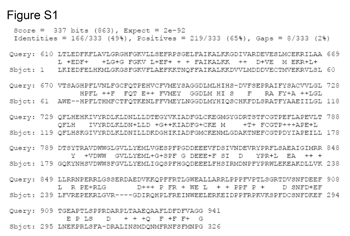

Supplement: Figure S1 — Sequence alignment between PKC-theta (Sbjct) and PRK1 (Query). (TIFF) [file pone.0034973.s001.tif]

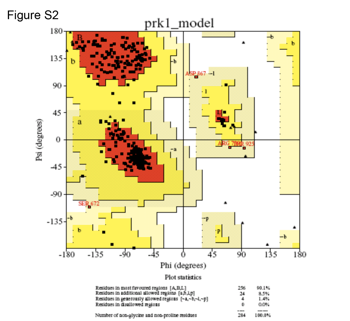

Supplement: Figure S2 — PROCHECK analysis. (TIFF) [file pone.0034973.s002.tif]

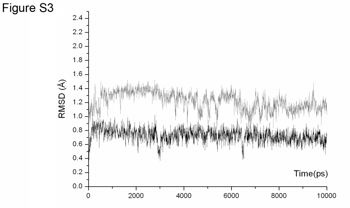

Supplement: Figure S3 — RMSD plot of the MD simulation of the PRK1-staurosporine complex using AMBER10. The grey line represents the RMSD plot for the PRK1 protein Cα-atoms, whereas the black line shows the fluctuation of the inhibitor structure. (TIFF) [file pone.0034973.s003.tif]

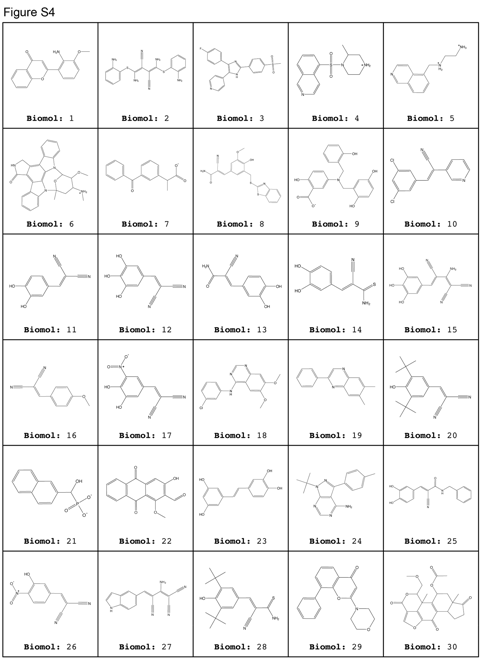

Supplement: Figure S4 — Compounds from the Biomol library, Compounds 1–30. (TIFF) [file pone.0034973.s004.tif]

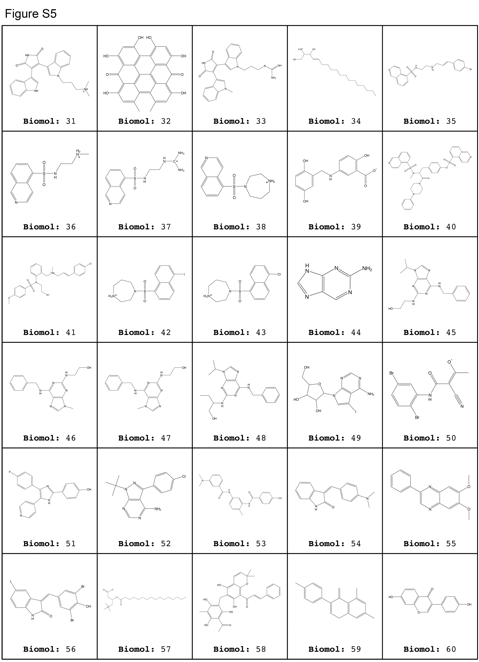

Supplement: Figure S5 — Compounds from the Biomol library, Compounds 31–60. (TIFF) [file pone.0034973.s005.tif]

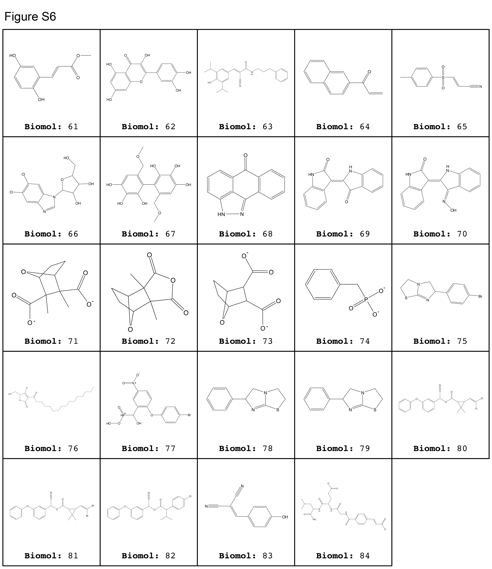

Supplement: Figure S6 — Compounds from the Biomol library, Compounds 61–84. (TIFF) [file pone.0034973.s006.tif]

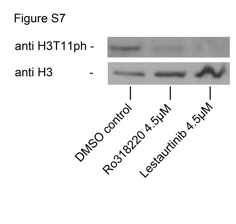

Supplement: Figure S7 — Effects of lestaurtinib and Ro318220 on the phosphorylation of H3T11 in LNCaP cells after 18 h. (TIFF) [file pone.0034973.s007.tif]
