# Supplementary material for: Lestaurtinib Inhibits Histone Phosphorylation and Androgen-Dependent Gene Expression in Prostate Cancer Cells
Source: PLoS One. 2012 Apr 20;7(4):e34973. doi: 10.1371/journal.pone.0034973 (PMC3332061; doi:10.1371/journal.pone.0034973)
Supplement: Table S1 — Scoring values obtained for 63 docked Biomol compounds and seven more of the tested kinase inhibitor. (DOC) [file pone.0034973.s008.doc]

**Supporting Information**

**Lestaurtinib Inhibits Histone Phosphorylation and Androgen- dependent Gene Expression in Prostate Cancer Cells**

Jens Köhler^1#^, German Erlenkamp^2^, Adrien Eberlin^3^, Tobias Rumpf,^1^ Inna Slynko,^2^ Eric Metzger^3^, Roland Schüle,^3^ Wolfgang Sippl,^2+^ and Manfred Jung**[[1]](#footnote-2)**^+^

^1^ J. Köhler, T. Rumpf, M. Jung; Albert-Ludwigs-University Freiburg, Institute of Pharmaceutical Sciences; Freiburg (Germany)

^2^ G. Erlenkamp, I. Slynko, W. Sippl; Martin-Luther University of Halle-Wittenberg, Department of Pharmaceutical Chemistry, Halle/Saale (Germany)

^3^ A. Eberlin, E. Metzger, R. Schüle; University of Freiburg Medical Center, Department of Urology/Women's Hospital and Center for Clinical Research, Freiburg (Germany)

^#^ Current address: Westfälische Wilhelms-University Münster, Institute of Pharmaceutical and Medicinal Chemistry, Münster (Germany)

# Table of Contents

Table S1 Scoring values obtaines for 63 docked Biomol compounds and seven more of the tested kinase inhibitor

## Tables

Table S1. Scoring values obtained for 63 docked Biomol compounds and seven more of the tested kinase inhibitors.

| **Cpd.** | **Chemscore**  **normalized** | **Goldscore**  **normalized** | **GBSA_score**  **normalized** | **Glidescore**  **normalized** | **P-score**  **normalized** | **Consensus**  **Score** |
| --- | --- | --- | --- | --- | --- | --- |
| 33 / Ro-318220 | 10.00 | 10.00 | 8.30 | 10.00 | 9.27 | 9.51 |
| 6 / Staurosporine | 9.76 | 7.14 | 10.00 | 9.27 | 9.98 | 9.23 |
| 31 | 9.93 | 8.13 | 7.45 | 9.56 | 9.14 | 8.84 |
| K252a | 7.34 | 8.68 | 6.93 | 7.27 | 9.91 | 8.03 |
| Lestaurtinib | 7.67 | 8.08 | 6.51 | 7.45 | 9.69 | 7.88 |
| SB216763 | 8.74 | 7.24 | 6.11 | 8.28 | 7.62 | 7.60 |
| 34 | 7.02 | 7.00 | 7.06 | 6.38 | 7.25 | 6.94 |
| 48 | 6.58 | 7.80 | 6.22 | 5.62 | 7.83 | 6.81 |
| 35 | 6.67 | 7.46 | 5.53 | 5.86 | 7.84 | 6.67 |
| 58 | 5.63 | 6.11 | 4.96 | 6.46 | 9.80 | 6.59 |
| 63 | 6.14 | 5.45 | 6.18 | 6.33 | 8.63 | 6.54 |
| Lapatinib | 6.50 | 6.79 | 6.70 | 3.19 | 9.30 | 6.50 |
| 41 | 5.61 | 4.91 | 5.66 | 6.55 | 9.51 | 6.45 |
| 9 | 7.79 | 6.24 | 3.94 | 5.39 | 8.50 | 6.37 |
| 8 | 7.53 | 3.86 | 5.42 | 5.87 | 8.16 | 6.17 |
| 69 | 7.30 | 5.66 | 4.58 | 6.67 | 6.57 | 6.16 |
| 45 | 5.81 | 7.38 | 5.30 | 5.01 | 7.15 | 6.13 |
| 62 | 5.16 | 6.64 | 5.28 | 6.38 | 7.14 | 6.12 |
| 22 | 7.47 | 5.50 | 4.51 | 6.42 | 6.63 | 6.11 |
| 84 | 4.46 | 7.39 | 3.52 | 5.16 | 10.00 | 6.10 |
| Erlotinib | 5.91 | 6.06 | 5.89 | 4.53 | 8.04 | 6.08 |
| 68 | 7.83 | 4.89 | 4.35 | 7.85 | 5.49 | 6.08 |
| 36 | 5.39 | 6.45 | 5.76 | 7.23 | 5.58 | 6.08 |
| 2 | 6.23 | 7.16 | 5.06 | 3.97 | 7.83 | 6.05 |
| 52 | 5.88 | 5.84 | 5.50 | 6.46 | 6.35 | 6.01 |
| Vatalanib | 6.14 | 4.96 | 6.79 | 4.70 | 7.41 | 6.00 |
| 35 | 7.16 | 6.87 | 7.78 | 0.00 | 8.11 | 5.98 |
| 47 | 5.64 | 6.96 | 5.43 | 5.25 | 6.63 | 5.98 |
| 24 | 5.75 | 5.88 | 5.32 | 6.45 | 6.48 | 5.98 |
| 38 | 4.59 | 5.62 | 6.90 | 6.48 | 6.19 | 5.96 |
| 56 | 4.97 | 6.26 | 6.63 | 5.56 | 6.30 | 5.94 |
| 76 | 6.50 | 6.70 | 5.78 | 2.35 | 8.10 | 5.89 |
| 51 | 5.29 | 5.53 | 5.21 | 5.26 | 7.46 | 5.75 |
| 46 | 5.00 | 7.13 | 5.11 | 4.95 | 6.51 | 5.74 |
| 23 | 5.49 | 5.45 | 4.83 | 6.62 | 6.22 | 5.72 |
| 32 | 7.16 | 6.48 | 6.40 | 0.00 | 8.49 | 5.71 |
| 42 | 5.55 | 5.10 | 6.20 | 5.24 | 6.44 | 5.71 |
| 39 | 6.49 | 6.45 | 3.30 | 5.60 | 6.53 | 5.67 |
| 37 | 4.36 | 6.33 | 4.52 | 6.84 | 6.17 | 5.64 |
| 3 | 5.39 | 4.45 | 4.41 | 5.30 | 8.16 | 5.54 |
| 18 | 6.10 | 4.63 | 4.88 | 5.54 | 6.50 | 5.53 |
| 25 | 6.04 | 5.74 | 2.76 | 6.13 | 6.93 | 5.52 |
| SB203580 | 5.67 | 4.63 | 5.14 | 4.07 | 7.97 | 5.50 |
| 4 | 4.65 | 4.71 | 4.94 | 6.95 | 6.16 | 5.48 |
| 5 | 5.02 | 5.00 | 4.57 | 7.39 | 5.09 | 5.41 |
| 1 | 4.94 | 4.44 | 4.60 | 6.56 | 6.40 | 5.39 |
| 77 | 3.88 | 7.61 | 4.18 | 4.65 | 6.58 | 5.38 |
| 67 | 4.39 | 5.98 | 3.96 | 4.91 | 7.14 | 5.28 |
| 28 | 5.94 | 4.37 | 4.56 | 4.68 | 6.81 | 5.27 |
| 43 | 4.36 | 4.40 | 5.04 | 6.01 | 6.47 | 5.25 |
| 60 | 3.78 | 4.04 | 3.53 | 8.39 | 6.29 | 5.21 |
| 54 | 4.54 | 3.64 | 4.14 | 7.27 | 6.35 | 5.19 |
| 66 | 5.09 | 5.13 | 2.83 | 6.30 | 6.53 | 5.18 |
| 14 | 5.53 | 5.42 | 3.58 | 6.11 | 5.00 | 5.13 |
| 70 | 6.90 | 7.30 | 4.70 | 0.00 | 6.72 | 5.12 |
| 49 | 3.56 | 5.88 | 3.12 | 6.40 | 6.54 | 5.10 |
| 15 | 4.00 | 6.14 | 4.50 | 4.67 | 6.18 | 5.10 |
| 53 | 4.82 | 3.72 | 4.13 | 4.66 | 8.02 | 5.07 |
| 27 | 4.10 | 5.84 | 4.32 | 4.83 | 6.21 | 5.06 |
| 13 | 5.83 | 4.74 | 3.99 | 5.29 | 4.98 | 4.97 |
| 26 | 5.07 | 4.52 | 3.55 | 4.99 | 5.14 | 4.65 |
| 21 | 5.42 | 5.72 | 0.90 | 5.62 | 5.30 | 4.59 |
| 17 | 5.32 | 4.25 | 3.70 | 4.03 | 5.44 | 4.55 |
| 61 | 4.47 | 5.27 | 3.00 | 4.92 | 4.90 | 4.51 |
| 83 | 3.11 | 7.74 | 2.80 | 4.50 | 4.32 | 4.49 |
| 20 | 3.60 | 2.80 | 4.52 | 4.54 | 6.49 | 4.39 |
| 11 | 4.70 | 4.39 | 2.83 | 4.70 | 4.56 | 4.24 |
| 12 | 4.14 | 4.32 | 2.53 | 5.07 | 4.95 | 4.20 |
| 44 | 3.35 | 4.30 | 2.58 | 6.53 | 3.35 | 4.02 |
| 50 | 3.68 | 4.15 | 3.27 | 0.00 | 5.21 | 3.26 |

1. Albert-Ludwigs-University Freiburg, Institute of Pharmaceutical Sciences; Albertstrasse 25, 79104 Freiburg (Germany); Tel: +49-761-203-4896; Fax: +49-761-203-6321

   Email: manfred.jung@pharmazie.uni-freiburg.de; ^+^Current additional affiliation

   Freiburg Institute of Advanced Studies (FRIAS), University Freiburg, Deutschland [↑](#footnote-ref-2)
